# *Bacillus* S-Layer-Mediated Innate Interactions During Endophthalmitis

**DOI:** 10.3389/fimmu.2020.00215

**Published:** 2020-02-12

**Authors:** Md Huzzatul Mursalin, Phillip S. Coburn, Erin Livingston, Frederick C. Miller, Roger Astley, Ana L. Flores-Mireles, Michelle C. Callegan

**Affiliations:** ^1^Department of Microbiology and Immunology, University of Oklahoma Health Sciences Center, Oklahoma City, OK, United States; ^2^Department of Ophthalmology, University of Oklahoma Health Sciences Center, Oklahoma City, OK, United States; ^3^Dean McGee Eye Institute, Oklahoma City, OK, United States; ^4^Department of Cell Biology and Department of Family and Preventive Medicine, University of Oklahoma Health Sciences Center, Oklahoma City, OK, United States; ^5^Department of Biological Sciences, University of Notre Dame, South Bend, IN, United States

**Keywords:** ocular infection, *Bacillus*, S-layer, immune response, inflammation, endophthalmitis, TLRs

## Abstract

*Bacillus* endophthalmitis is a severe intraocular infection. Hallmarks of *Bacillus* endophthalmitis include robust inflammation and rapid loss of vision. We reported that the absence of *Bacillus* surface layer protein (SLP) significantly blunted endophthalmitis severity. Here, we further investigated SLP in the context of *Bacillus-*retinal cell interactions and innate immune pathways to explore the mechanisms by which SLP contributes to intraocular inflammation. We compared phenotypes of Wild-type (WT) and SLP deficient *(*Δ*slpA*) *Bacillus thuringiensis* by analyzing bacterial adherence to and phagocytosis by human retinal Muller cells and phagocytosis by mouse neutrophils. Innate immune receptor activation by the *Bacillus* envelope and purified SLP was analyzed using TLR2/4 reporter cell lines. A synthetic TLR2/4 inhibitor was used as a control for this receptor activation. To induce endophthalmitis, mouse eyes were injected intravitreally with 100 CFU WT or Δ*slpA B. thuringiensis*. A group of WT infected mice was treated intravitreally with a TLR2/4 inhibitor at 4 h postinfection. At 10 h postinfection, infected eyes were analyzed for viable bacteria, inflammation, and retinal function. We observed that *B. thuringiensis* SLPs contributed to retinal Muller cell adherence, and protected this pathogen from Muller cell- and neutrophil-mediated phagocytosis. We found that *B. thuringiensis* envelope activated TLR2 and, surprisingly, TLR4, suggesting the presence of a surface-associated TLR4 agonist in *Bacillus*. Further investigation showed that purified SLP from *B. thuringiensis* activated TLR4, as well as TLR2 *in vitro*. Growth of WT *B. thuringiensis* was significantly higher and caused greater inflammation in untreated eyes than in eyes treated with the TLR2/4 inhibitor. Retinal function analysis also showed greater retention of A-wave and B-wave function in infected eyes treated with the TLR2/4 inhibitor. The TLR2/4 inhibitor was not antibacterial *in vitro*, and did not cause inflammation when injected into uninfected eyes. Taken together, these results suggest a potential role for *Bacillus* SLP in host-bacterial interactions, as well as in endophthalmitis pathogenesis via TLR2- and TLR4-mediated pathways.

## Introduction

Endophthalmitis is a microbial infection of the posterior segment of the eye (1–6). Microbes can enter this part of the eye following a penetrating injury to the globe (post-traumatic), surgery or intraocular injection (post-operative), or following hematogenous spread from another infection site (endogenous) (7–15). Hallmarks of this disease include intraocular inflammation and retinal damage, resulting in some degree of vision loss. Unfortunately, blindness can occur and removal of the globe may be necessary, even when prompt and aggressive therapeutic measures are taken (5, 16–19). Endophthalmitis caused by *Bacillus* spp is more devastating compared to endophthalmitis caused by other bacterial pathogens associated with this disease (7, 20). Among members of the *Bacillus cereus sensu lato* group (comprised of *Bacillus anthracis, Bacillus cereus*, and *Bacillus thuringiensis*), only *B. cereus* and *B. thuringiensis* have been reported as the causative agents of intraocular infection (21–25). Significant vision loss has been reported to occur in the majority of *Bacillus* endophthalmitis cases, with half of those devastating cases resulting in removal of the globe (enucleation) (26–32). *Bacillus* endophthalmitis is indeed a medical emergency, and its rapid and severe course requires immediate therapeutic attention to prevent further deterioration of the eye (33–36). At present, there is no consistently effective therapeutic strategy which mitigates vision loss during severe cases of endophthalmitis, including those caused by *B. cereus* (16, 17, 37–41). The practice of adding anti-inflammatory agents to antibiotics has not proven effective in arresting inflammation and vision loss (42–45). It is clear that other therapeutic strategies are needed to prevent the sight-threatening consequences of this infection.

*B. cereus* spp are Gram-positive, motile, β-hemolytic, spore-forming rods, and are widely disseminated in nature (23, 24). We reported that the *Bacillus cereus* cell wall, and secreted toxins and proteases contributed to the pathogenicity of experimental endophthalmitis (5, 19, 46, 47). The PlcR quorum sensing system controls the synchronized synthesis of a majority of these extracellular virulence factors and is therefore important in *Bacillus* intraocular virulence (48–51). The absence of individual *B. cereus* toxins did not blunt intraocular virulence (19, 47). However, in the absence of PlcR, we observed delayed evolution, but not complete attenuation of *Bacillus* endophthalmitis, suggesting the contribution of the bacterial cell wall or other components to this disease (49).

We reported that metabolically inactive *B. cereus* triggered robust intraocular inflammation, suggesting that cell wall components contribute to the activation of pro-inflammatory pathways (5). *B. cereus* have an architecturally unique envelope. In addition to peptidoglycan, lipoteichoic acid, and lipoproteins, which are all common among Gram-positive ocular pathogens, the envelope of some *B. cereus* has flagella and a paracrystalline surface protein called the S-layer protein (SLP) (52–56). Structurally, SLPs are widely diverse among species and sequence similarities from different species are low.

Since SLPs are major surface antigens, the contributions of SLPs to microbial pathogenesis have been studied in some model organisms (56–63). As the outermost layer of the surface of some bacterial strains, SLPs promote adherence of bacteria to cell membranes and extracellular matrix components, and also contribute to biofilm formation (64–68). SLPs also act as barrier, protecting bacteria from complement-mediated phagocytosis and killing (69–72). A recent report from our laboratory demonstrated that the absence of *Bacillus* SlpA significantly reduced endophthalmitis disease severity in mice (73). We also demonstrated that *Bacillus* SLP preparations activated nuclear factor kappa-light-chain-enhancer of activated B cells (NF-κB) and induced the expression of inflammatory mediators from retinal cells (73). However, the underlying mechanisms by which SLPs impact endophthalmitis pathogenesis remains unclear.

The ocular environment is immune privileged, and its inner tissues contain different types of cells that not only maintain the structural integrity and homeostasis of this tissue, but also act as innate immune cells which express several innate receptors (74–81). During endophthalmitis, TLRs on retinal cells sense invading microbes and induce the production of inflammatory mediators, which leads to recruitment of polymorphonuclear neutrophils (PMN) into the eye (79, 82–84). Almost all TLRs signal through the myeloid differentiation primary response gene-88 (MyD88) dependent pathway. In addition to MyD88 pathway, TLR4 can mediate signaling through the Toll/interleukin-1 receptor (TIR) domain containing adaptor-inducing interferon-β (TRIF) pathway (85–88). We reported that the inflammatory response in *Bacillus* endophthalmitis is primarily facilitated through TLR2 and TLR4, but not through TLR5 (89–91). The absence of TLR2 or TLR4 resulted in less PMN infiltration, inflammatory mediator production, and pathological damage during *Bacillus* endophthalmitis (90, 91). Blocking TLRs in this disease may effectively blunt inflammation. Identifying *B. cereus* ligands that trigger these innate pathways may help us to more clearly understand the pathological events of this disease.

*Bacillus* endophthalmitis is at or near the top of the list of rapidly blinding ocular diseases, but the level of understanding of the host/pathogen relationship in this disease is fairly limited. The earliest host response in *Bacillus* endophthalmitis is the activation of TLRs that drive the intense intraocular inflammation. Since SLPs activated NF-κB and triggered the production of proinflammatory mediators in human retinal Muller cells, we hypothesized that *B. cereus* SLPs initiate early events in endophthalmitis pathogenesis through interactions with retinal cells and by activating innate pathways. Results from this study will broaden our understanding about the mechanisms of early and potentially damaging immune response and may aid in the development of potential therapeutics to prevent inflammation and vision loss during *Bacillus* endophthalmitis.

## Materials and Methods

### Bacterial Strains

*B. thuringiensis subsp. galleriae* NRRL 4045 (WT) or its isogenic SLP deficient mutant (Δ*slpA*) (73, 92) were used to initiate experimental endophthalmitis in mice, as previously described (89–91, 93–98). *Staphylococcus aureus* strain 8325-4, *Enterococcus faecalis* strain E99, *Staphylococcus epidermidis* ATCC 12228, and *Streptococcus pneumoniae* strain TIGR4 were used for the preparation of bacterial cell envelopes.

### Bacterial Adherence Assay

To quantify bacterial attachment to human retinal Muller cells (MIO-M1; a kind gift from Dr. Astrid Limb, UCL Institute of Ophthalmology, London), human retinal pigment epithelial cells (ARPE-19; American Type Culture Collection, Manassas, VA), and retinal photoreceptor-like 661W cells (99), we used an aerobic bacterial adherence assay. Immortalized human retinal pigmented epithelial (ARPE-19) and Muller cells were cultured in Dulbecco's Modified Eagle's Medium (DMEM)/F-12 (GIBCO, Grand Island, NY) supplemented with 10% fetal bovine serum (FBS, Sigma Aldrich, St. Louis MO) and 1% Pen Strep (GIBCO) Retinal photoreceptor-like 661W cells were cultured in DMEM containing GlutaMAX^TM^-l (GIBCO), supplemented with 10% (v/v) FBS (100–103). All cells were maintained in a humidified 5% CO_2_ incubator at 37°C.

Confluent monolayers of each of these cell types (~2 × 10^6^ cells) were grown in 6-well plates, and transferred to antibiotic- and serum-free DMEM 6 h prior to performing the adherence assay. Overnight cultures of WT and Δ*slpA B. thuringiensis* were harvested by centrifugation and washed twice with DMEM to exclude the effects of secreted proteins, including any toxins. Antibiotic- and serum-free medium were removed from the cells, and bacteria were added to the wells at a multiplicity of infection (MOI) of 20 in a total volume of 2 ml DMEM. Equal numbers of WT and Δ*slpA B. thuringiensis* bacteria were added to cell free wells as controls. Cell-free controls were used to verify whether bacteria adhered to the plastic surface of the six- well plates. After a 40 min incubation in a humidified 5% CO_2_ incubator at 37°C, retinal cells and adherent bacteria were washed twice with PBS. Adherent cells were then removed with a tissue cell scraper, vortexed, and serially diluted to quantify the adherent bacteria. The percent of adherent bacterial cells was calculated as the ratio of recovered bacteria to input bacteria multiplied by 100 (63, 66, 67, 104).

### Isolation of Primary Neutrophils From Mice

Primary neutrophils were collected from mouse bone marrow by using a neutrophil isolation kit (MACS, Miltnyl Biotech, Gladbach, Germany) according to the manufacturer's instructions. Femurs were harvested from adult C57BL/6J mice. Bone marrow was collected in a 50 mL Falcon tube containing RPMI media (GIBCO) with 10% FBS (Sigma Aldrich) using a 10 ml syringe. The bone marrow was then centrifuged and washed with wash buffer (PBS, pH 7.2, 0.5% bovine serum albumin (BSA), and 2 mM EDTA). Cells were counted using a hemocytometer. For every 5 × 10^7^ total cells, 200 μL of wash buffer and 50 μL neutrophil biotin-antibody cocktail were added. Cells were mixed and incubated for 10 min at 4°C. Cells were then washed and the pellet was resuspended in 400 μL of wash buffer and 100 μL of anti-biotin microbeads. Cells were mixed and incubated at 4°C. After 15 min, cells were washed and resuspended to 10^8^ cells in 500 μL of buffer. For magnetic separation, an appropriate MACS column and separator were chosen according to the number of total cells and number of neutrophils. The LS column was used and placed inside a MACS separator. A 15 mL tube was placed under the column and the column was washed with 3 mL of wash buffer. When the wash buffer was completely removed, the 15 mL tube was replaced with a new one. The total sample (500 μL) was then loaded onto the column, and 3 mL of wash buffer was added 3 times onto the column and cells were collected. Cells were counted and centrifuged at 100 × *g* for 10 min, and resuspended in RPMI medium (96, 105, 106). One group of isolated cells was then immunolabeled with Ly6G and CD11 antibodies, washed, and fixed as previously described (107). Samples were analyzed using a MacsQuant flow cytometer and MacsQuantify software (Miltenyi Biotec). Neutrophil purity in each isolation was ~85.6%.

### Bacterial Phagocytosis Assay

Human retinal Muller cells (MIO-M1), neutrophil like HL-60 cells, and mouse primary neutrophils were used in a gentamicin exclusion assay to assess the impact of SLPs on phagocytosis. Undifferentiated HL-60 cells were differentiated into neutrophil like-cells by adding 1.3% DMSO for 6 days (108–110). After 6 days, cells had neutrophil-like morphology, as confirmed by microscopy (5). Approximately 1 × 10^5^ of these cells were incubated at 20 MOI (~2 × 10^6^) with WT or Δ*slpA B. thuringiensis* for 90 min. One group of cells was washed and treated with 200 μg/mL gentamicin for 60 min to kill all extracellular bacteria, and another group of cells was centrifuged, washed and lysed with 0.5% Triton X-100. This later group contained intra- and extracellular bacteria. After 60 min, the gentamicin-treated cells were centrifuged, washed to remove the residual antibiotic, and lysed with 0.5% Triton X-100. This group represented only the intracellular bacteria. Equal numbers of WT and Δ*slpA B. thuringiensis* (~2 × 10^6^ in 2 mL) were incubated with 200 μg/mL gentamicin for 60 min and used as a control (96, 104, 111). CFU were enumerated by serial dilution and plating.

### Preparation of Bacterial Cell Envelopes

*B. thuringiensis subsp. galleriae* NRRL 4045 (WT) and its isogenic SLP deficient mutant (Δ*slpA*), *S. aureus* 8325-4, *E. faecalis* E99, and *S. epidermidis* strain ATCC 12228 were each grown for 18 h at 37°C in brain heart infusion (BHI; VWR, Radnor PA) broth and 20 μl aliquots were removed, serially diluted, and plated to quantify bacteria. *S. pneumonia* was grown in Todd Hewitt Broth (THB; VWR) with 0.5% yeast extract and also grown for 18 h at 37°C. Cultures were harvested by centrifugation at 3,000 × *g* for 15 min at 4°C, and washed twice with PBS (pH 7.4) in endotoxin free water (HyPure cell culture grade water, GE Healthcare Life Science, Logan UT). Pellets were resuspended with equal volumes of PBS and heat inactivated at 65°C for 15 min. Sterility was tested by spread plating an aliquot of each culture onto a BHI agar plate. Cells were then centrifuged at 3,000 × *g* for 15 min, and pellets were washed twice with equal volumes of PBS. The bacterial pellets were then lyophilized, resuspended with equal volumes of PBS, and diluted to the required concentrations for use in the TLR2 and TLR4 reporter assays (5, 73, 112).

### Purification of *Bacillus* SLP

WT and Δ*slpA B. thuringiensis* were grown for 18 h at 37°C in BHI, harvested by centrifugation at 3,000 × *g* for 15 min at 4°C, and washed twice with chilled HyPure cell culture grade water (GE Healthcare Life Science). As previously described, pellets were then resuspended in 1/10th of the initial volume of 3M guanidine hydrochloride (GHCL; pH 2.5; Sigma Aldrich) and incubated at 37°C for 1 h. The extracted SLP was separated from the pellets by centrifugation at 18,000 × *g* at 4°C for 15 min. Supernatants were dialyzed (Pur-A-Lyzer^TM^ 50kDa dialysis kit, Sigma Aldrich) against 2L of tris/HCL (pH 8.0; Research Products International Corporation, Mt. Prospect, IL) at 4°C for 24 h with four exchanges of dialysis buffer to remove residual GHCL. Protein concentrations were quantified by bicinchoninic acid assay (Sigma Aldrich) according to the manufacturer's instructions. Endotoxin levels were quantified using the Pierce LAL chromogenic endotoxin kit (ThermoFisher Scientific, MA) according to the manufacturer's instructions. Purity was confirmed by PAGE and Coomassie staining, as previously described (73, 92).

### TLR2/TLR4 Reporter Assay

HEK-Blue^TM^ cells were purchased from Invivogen (San Diego, CA) and used as previously described (73). HEK-Blue™ hTLR2 and HEK-Blue™ hTLR4 were used for the recognition of TLR2 and TLR4 agonists, respectively. hTLR2 and hTLR4 cells were cultured (up to 20 passages) in DMEM containing GlutaMAX^TM^-l (GIBCO), supplemented with 10% (v/v) FBS (Sigma Aldrich) and HEK-Blue Selection antibiotics (Invivogen) in a humidified 5% CO_2_ incubator at 37°C. hTLR2 and hTLR4 reporter cell lines were treated with bacterial envelopes, or SLP fractions from WT or Δ*slpA B. thuringiensis* with or without the synthetic TLR2/4 inhibitor OxPAPC (Invivogen) to assess receptor activation/inhibition (73, 89).

*B. thuringiensis, S. aureus, S. epidermidis* at 10^6^ envelopes/20 μl, and *E. faecalis* and *S. pneumoniae* at 10^8^ envelopes/20 μl were used to assess TLR2/4 activation. The envelope inoculum number was determined based on the equivalent number of viable organisms present during early infection (5). To measure the TLR2/4 activation by purified SLP, 10 μg/ml SLP from WT *B. thuringiensis* was used. The SLP fraction from Δ*slpA B. thuringiensis* was used as an extract control. In both assays, Pam3Csk4 (0.25 ng/mL; Invivogen) was used as a positive control for the hTLR2, and a negative control for the hTLR4 reporter assays. LPS (100 ng/mL; Invivogen) was used as a positive control for the hTLR4, and a negative control for the hTLR2 reporter assays. Endotoxin free water (GE Healthcare Life Science) was used as a negative control for both hTLR2 and hTLR4 reporter assays. To inhibit TLR2/4 activation, an oxidized phospholipid OxPAPC (0.15μg/μL) was used with Pam3Csk4, LPS, and purified SLP (113). Samples, controls, and inhibitors (20 μL) were added to appropriate wells of 96-well plates. hTLR2 and hTLR4 reporter cells at 50 to 80% confluency were washed with pre-warmed PBS (pH 7.4; GIBCO). After detaching the cells with PBS, hTLR2 cells were resuspended to 5.0 × 10^4^ and hTLR4 cells to 2.5 × 10^4^ in 180 μl of HEK-Blue^TM^ Detection medium (Invivogen). For the OxPAPC-treated groups, 5.0 × 10^4^ /160 μl hTLR2 and 2.5 × 10^4^/160 μl hTLR4 cell suspensions were prepared. Each cell suspension was immediately added into the appropriate wells of the 96-well plates, and incubated for 14 h at 37°C in 5% CO_2_. Activation of TLR2 and TLR4 (production of SEAP) was measured using a spectrophotometer at 620-655nm. TLR2/4 activation was presented as percent of TLR2/4 activation relative to the positive controls Pam3Csk4 and LPS (73, 89, 114).

### Mice and Intraocular Infection

All *in vivo* experiments were performed with C57BL/6J mice purchased from Jackson Labs (Bar Harbor ME, Stock No. 000664). Mice were housed on a 12 h on/12 h off light cycle in biohazard level 2 conditions and acclimated for at least 2 weeks to equilibrate their microbiota. Mice were 8–10 weeks of age at the time of the experiments. Mice were sedated using a combination of ketamine (85 mg/kg body weight; Ketathesia, Henry Schein Animal Health, Dublin, OH) and xylazine (14 mg/kg body weight; AnaSed, Akorn Inc., Decatur, IL). Four groups of C57BL/6J mice were used in this experiment. The first two groups of mice were infected with 100 CFU WT *B. thuringiensis*/0.5 μl BHI, and the third group was infected with 100 CFU Δ*slpA B. thuringiensis*/0.5 μl BHI into the right eye using a sterile glass capillary needle, as previously described (73, 89–91, 93, 95–98). The fourth group was not infected. At 4 h postinfection, the second group of infected mice and fourth group of uninfected mice were intravitreally treated with 30 ng/μL OxPAPC. At 10 h postinfection, electroretinography was performed prior to euthanasia by CO_2_ inhalation, and then eyes were harvested for quantitation of viable intraocular bacteria, retinal function, and PMN infiltration, and analysis of ocular architecture by histology, as described below.

### Intraocular Bacterial Quantitation

As previously described (73, 89–91, 93, 94, 96–98), eyes were harvested from euthanized mice at specific time points, homogenized in 400 μl PBS with sterile 1-mm glass beads (BioSpec Products, Inc., Bartlesville OK), serially diluted 10-fold in PBS, and plated onto BHI agar plates.

For *in vivo* bacterial growth analysis at different time points, experimental endophthalmitis was induced by intravitreally injecting approximately 100 CFU WT *B. thuringiensis* in 0.5 μl BHI into the right eyes of mice. At 4 h postinfection, one group of infected eyes was treated with OxPAPC, and another group served as the untreated control. At 2 h intervals thereafter, eyes were harvested for quantitation of intraocular bacterial growth (73, 89–91, 93, 94, 96–98).

### *In vitro* Bacterial Quantitation

Potential antimicrobial activity of OxPAPC was assessed *in vitro*. WT *B. thuringiensis* was cultured for 18 h at 37°C with aeration in BHI medium. The culture was then diluted to 10^3^ CFU/mL in fresh BHI containing 0.1, 1, or 10 μg/mL OxPAPC, and incubated for 18 h at 37°C. At 2 h intervals during this period, 20 μl aliquots were serially diluted 10-fold in PBS, and plated onto BHI agar plates (73, 93).

### Retinal Function Analysis

Electroretinography (ERG) was used to quantify retinal function as previously described (5, 47, 51, 73, 91, 93, 94, 96, 97) in *Bacillus-*infected and OxPAPC-treated eyes. After infection/treatment, mice were dark adapted for 6 h. Mice were then anesthetized as described above, and pupils were dilated with topical phenylephrine (Akorn, Inc., IL). Two gold wire electrodes were placed onto each cornea. Reference electrodes were attached to the tail and forehead. Eyes were then stimulated by five flashes of white light (1,200 cd s/m^2^) and retinal responses were recorded as A-wave (retinal photoreceptor cell function) and B-wave (bipolar cell, Muller cell, and second order neuronal function) amplitudes for infected eyes and compared with the uninfected eyes of the same animal (Espion E2 software, Diagnosys LLC, Lowell MA) (5, 47, 51, 73, 91, 93, 94, 96, 97).

### Histology

Infected/treated eyes were harvested from euthanized mice at 10 h postinfection. Harvested eyes were incubated in High Alcoholic Prefer fixative for 30 min, and then transferred to 70% ethanol. Paraffin-embedded eyes were sectioned and stained with hematoxylin and eosin (H&E) (73, 89, 90, 94, 96–98).

### Inflammatory Cell Influx

Inflammatory cell infiltration was estimated by quantifying myeloperoxidase (MPO) using a sandwich ELISA (Hycult Biotech, Plymouth Meeting PA), as previously described. At 10 h postinfection, eyes were harvested, transferred into PBS supplemented with proteinase inhibitor cocktail (Roche Diagnostics, Indianapolis, IN) and homogenized using 1-mm sterile glass beads (BioSpec Products, Inc.). Uninfected eye homogenates were the negative controls. The lower limit of detection for this assay was 2 ng/ml (73, 89, 91, 96–98).

### Statistics

GraphPad Prism 7 was used for the statistical analysis (Graph-Pad Software, Inc., La Jolla, CA). Mann-Whitney *U*-test was used for statistical comparisons unless otherwise specified. *P*-values of <0.05 were considered significant (93, 94, 96, 115). For all assays, *N*-values represented single biological replicates.

## Results

### SlpA Contributes to the Adherence of *Bacillus* to Retinal Cells

**Figure 1:**
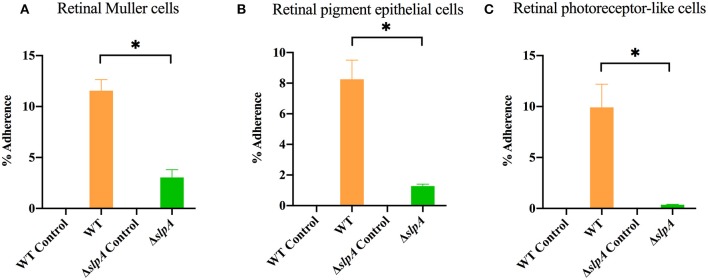
*Bacillus* SLP contributes to adherence to retinal cells *in vitro*. Three different retinal cell types were incubated with WT *B. thuringiensis* or Δ*slpA B. thuringiensis* for 40 min to assess bacterial adherence. Compared to WT, Δ*slpA B. thuringiensis* demonstrated a significant reduction in adherence to **(A)** human retinal Muller MIO-M1 cells, **(B)** human retinal pigment epithelial (ARPE-19) cells, and **(C)** retinal photoreceptor-like 661W cells. WT and Δ*slpA B. thuringiensis* in cell-free wells served as controls. Values represent the mean ± SEM of *N* ≥ 5 for at least two separate experiments; *P < 0.05.

As *Bacillus* migrate within the posterior segment of the eye, organisms physically interact with retinal cells (5, 73). The first step in this interaction is adherence, and we hypothesized that SLPs mediated that interaction. An experiment to evaluate the role of SlpA in bacterial attachment to retinal cells is depicted in Figure 1. In the absence of SlpA, significant reductions in percent Δ*slpA B. thuringiensis* adherence were seen with human retinal Muller MIO-M1 cells (*P* < 0.0001, Figure 1A), retinal pigment epithelial cells (ARPE-19) (*P* = 0.0022, Figure 1B), and retinal photoreceptor-like 661W cells (*P* = 0.0022, Figure 1C) compared to that of WT *B. thuringiensis*. No bacteria were recovered from cell-free controls, suggesting no adherence to the plastic surface of the wells. These findings demonstrated that SlpA contributed to bacterial adherence to retinal cells, suggesting that SLPs may play a role in bacterial adherence to retinal cells during the early stage of *Bacillus* endophthalmitis.

### SlpA Protects *Bacillus* From Phagocytosis

**Figure 2:**
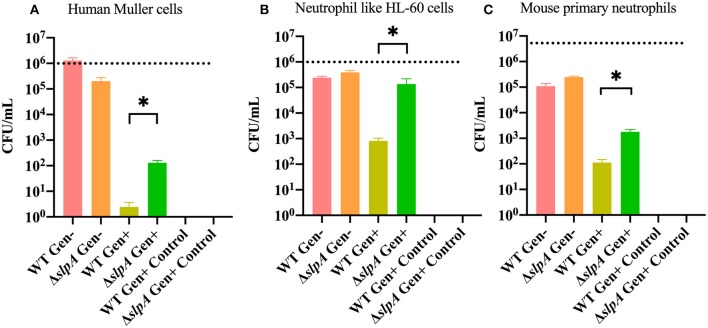
*Bacillus* SLP provides protection from phagocytosis. Muller cells, neutrophil-like HL60 cells, and mouse primary neutrophils were incubated with WT or Δ*slpA B. thuringiensis* for 90 min. Cells were then treated with gentamicin for 60 min to kill external bacteria. Compared to WT *B. thuringiensis*, internalization of Δ*slpA B. thuringiensis* was significantly greater by **(A)** human retinal Muller cells, **(B)** neutrophil-like HL-60 cells, and **(C)** mouse primary neutrophils. Gen+, Gentamicin treated; Gen-, Gentamicin untreated. Values represent mean ± SEM of *N* ≥ 5 for at least two separate experiments; *P < 0.05. Dashed lines represent the initial bacterial inoculum.

Interactions between *Bacillus* and retinal and immune cells may be important in initiating the subsequent immune response. A gentamicin (Gen) exclusion phagocytosis assay was used to determine the role of SlpA in internalization by human retinal Muller cells (MIO-M1), neutrophil like HL-60 cells, and mouse primary neutrophils (Figure 2). Significant increases in internalization of Δ*slpA B. thuringiensis* were seen with human retinal Muller cells (*P* = 0.0122, Figure 2A), neutrophil like HL-60 cells (*P* = 0.0049, Figure 2B), and mouse primary neutrophils (*P* = 0.0002, Figure 2C) compared to that of WT *B. thuringiensis*. No bacteria were recovered after the incubation with gentamicin, indicating that WT and Δ*slpA B. thuringiensis* were susceptible to the antibiotic. Taken together, these results demonstrated that SlpA directly interfered with internalization by human retinal Muller cells and professional phagocytic cells, suggesting that SLP may protect the pathogen from phagocytosis during active infection.

### *Bacillus* Envelope Contains an Unexpected TLR4 Agonist

**Figure 3:**
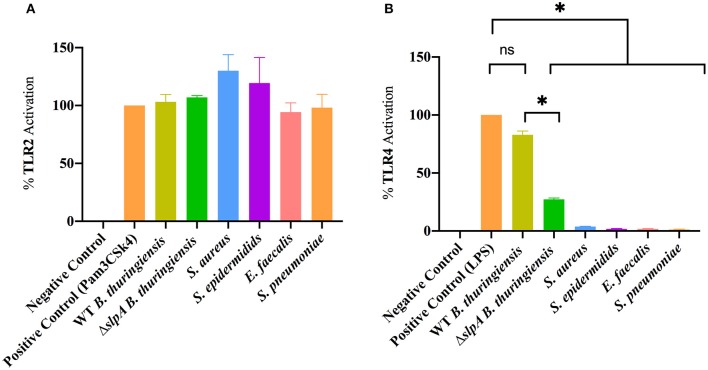
*Bacillus* envelope possesses an unexpected TLR4 agonist. HEK-Blue™ hTLR2 and hTLR4 reporter cells were incubated with envelope preparations of WT or Δ*slpA B. thuringiensis, S. aureus, S. epidermidis, E. faecalis*, or *S. pneumoniae* for 14 h at 37°C in 5% CO_2_ **(A)** The envelopes of WT *and* Δ*slpA B. thuringiensis, S. aureus, S. epidermidis, E. faecalis*, and *S. pneumoniae* activated TLR2. **(B)** Among the five Gram-positive endophthalmitis pathogens, only the envelopes of WT *B. thuringiensis* activated TLR4. Values represent mean ± SEM of *N* ≥ 4 for at least two separate experiments; *P < 0.05.

Mice which lack functional TLR2 or TLR4 have a reduced intraocular inflammatory response upon intravitreal challenge with *Bacillus* (90, 91), suggesting that this organism interacts with those receptors. Here, we investigated whether the envelopes of common Gram-positive endophthalmitis pathogens (WT and Δ*slpA B. thuringiensis, S. aureus, S. epidermidis, E. faecalis*, and *S. pneumoniae*) activated TLR2 and TLR4 in hTLR2 or hTLR4 reporter cell line assays (Figure 3). Envelope preparations from all five species activated TLR2 (Figure 3A). Surprisingly, only WT *B. thuringiensis* envelopes significantly activated TLR4 (*P* = 0.0286), whereas other Gram-positive endophthalmitis pathogens did not (Figure 3B). Activation of TLR4 was significantly higher (*P* = 0.0286) in WT *B. thuringiensis* than Δ*slpA*. These results suggest that the *Bacillus* envelope possesses universal TLR2 agonists and one or more unexpected TLR4 agonists.

### SLP of *Bacillus* Is Necessary for Activation of Both TLR2 and TLR4

**Figure 4:**
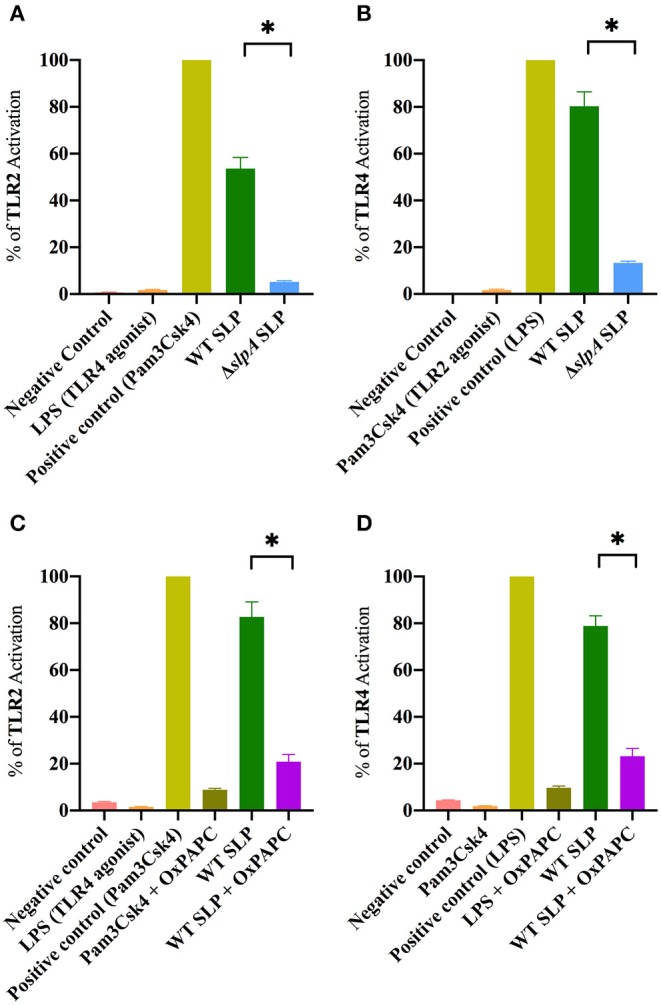
*Bacillus* SLP is a potent activator of TLR2 and TLR4. **(A)** SLP from WT *B. thuringiensis* activated TLR2 to a significantly greater degree than did the extract control (Δ*slpA*). LPS, a TLR4 agonist, was used as a negative control. **(B)** WT SLP significantly activated TLR4 compared to the extract control (Δ*slpA*). Pam3Csk4, a TLR2 agonist, was used as a negative control. **(C)** Treatment with OxPAPC significantly inhibited TLR2 activation by the positive control Pam3Csk4 and WT SLP. **(D)** Treatment with OxPAPC significantly inhibited TLR4 activation by the positive control LPS and WT SLP. Values represent mean ± SEM of *N* ≥ 5 for at least two separate experiments; *P < 0.05.

SLP from WT *B. thuringiensis* induced inflammatory mediator expression from retinal Muller cells by activating the canonical NF-κB pathway (73). Since the envelope of *B. thuringiensis* activated TLR2/4, we next determined whether its SLP activated TLR2 and TLR4 in similar assays. Purified SLP from WT *B. thuringiensis* activated TLR2 to a significantly higher degree than the extract control from Δ*slpA B. thuringiensis* (*P* = 0.0003; Figure 4A). Purified SLP from *B. thuringiensis* also significantly activated TLR4 to a greater degree than did the extract control from Δ*slpA B. thuringiensis* (*P* = 0.0003; Figure 4B). To further evaluate the activation of TLR2 and TLR4 by SLP, we included an oxidized phospholipid (OxPAPC) in the reporter assay to inhibit the activation of both TLR2 and TLR4. OxPAPC significantly inhibited TLR2 activation by the TLR2 agonist Pam3Csk4 and by purified SLP from WT *B. thuringiensis* (*P* = 0.0022; Figure 4C). OxPAPC also significantly reduced the activation of TLR4 by LPS and by purified SLP (*P* = 0.0022; Figure 4D) SLP-mediated TLR2 and TLR4 activation in OxPAPC treated groups were 74.7 and 70.7% lower than TLR2 or TLR4 activation in the untreated groups, respectively. Together these findings demonstrated that SLPs not only activated TLR2, but also TLR4. This suggests that SLP is a potent stimulator of both TLR2 and TLR4 innate pathways, and may contribute to the production of inflammatory mediators during experimental endophthalmitis.

### Inhibition of TLR2/4 Activation Resulted in Reduced Bacterial Burden During Experimental *Bacillus* Endophthalmitis

**Figure 5:**
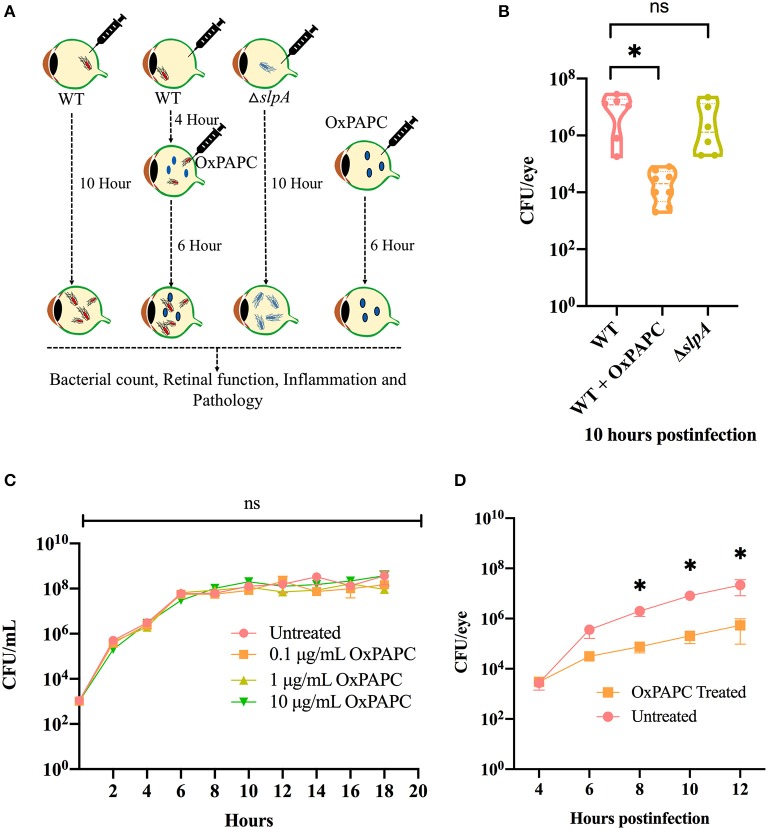
OxPAPC treatment resulted in reduced intraocular bacterial load during *Bacillus* endophthalmitis. **(A)** Experimental design of *in vivo* inhibition of TLR2/4 activation. **(B)** C57BL/6J mouse eyes were infected with 100 CFU WT or Δ*slpA B. thuringiensis*. At 4 h postinfection WT infected eyes were treated with 30 ng/μL OxPAPC. At 10 h postinfection eyes were harvested, homogenized, and analyzed for bacterial growth. Compared to untreated WT-infected C57BL/6J mouse eyes, a significant reduction in bacterial burden was observed in the OxPAPC-treated group at 10 h postinfection. No difference in intraocular bacterial count was observed between WT and Δ*slpA* infected eyes at 10 h postinfection. Values represent mean ± SEM of log10 CFU/eye of *N* ≥ 5 eyes for at least two separate experiments. **(C)** The *in vitro* growth of WT *B. thuringiensis* in BHI was not affected by the presence of 0.1, 1, or 10 μg/mL OxPAPC. Values represent the mean ± SEM of *N* = 3; multiple comparison, 2-way ANOVA **(D)** Treatment of eyes with OxPAPC 4 h after infection with 100 CFU of WT *B. thuringiensis* resulted in decreased bacterial growth. Data represent the mean ± SEM of log10 CFU/eye of *N* ≥ 5 eyes per time point for at least two separate experiments; ns: *P* > 0.05, *P < 0.05 at all time points.

There were no changes the intraocular bacterial burden in TLR2^−/−^ or TLR4^−/−^ mice infected with *B. cereus* (90, 91). Here, we investigated whether inhibition of both TLR2 and TLR4 activation affected bacterial growth during experimental endophthalmitis. Figure 5A depicts the experimental design. Inhibition of the TLR2/4 pathways by OxPAPC significantly reduced the bacterial load in WT infected mouse eyes relative to that of untreated eyes (*P* = 0.0007; Figure 5B) at 10 h postinfection. There was no difference in bacterial load observed between WT and Δ*slpA* infected mouse eyes (*P* = 0.3680; Figure 5B). At this time, the growth rates of WT and Δ*slpA B. thuringiensis* infected eyes (2.2 and 1.9 h^−1^) were faster than that in the WT-infected and OxPAPC treated eyes (0.76 h^−1^). To determine whether OxPAPC possessed bactericidal activity, we analyzed WT *B. thuringiensis* growth in the presence of increasing concentrations (0.1, 1, and 10 μg/mL) of OxPAPC. As shown in Figure 5C, OxPAPC did not alter bacterial growth *in vitro* at any of the concentrations tested. To investigate whether this phenomenon of reduced bacterial load only occurred *in vivo*, we assessed bacterial growth at varying time points after infection after treatment with OxPAPC and observed that bacterial concentrations were significantly lower in OxPAPC treated groups at 8 h (*P* = 0.0260), 10 h (*P* = 0.0043) and 12 h (*P* = 0.0152) postinfection (Figure 5D). Taken together, these findings demonstrated that inhibition of TLR2/4 activation contributed to reduced bacterial burden during experimental *Bacillus* endophthalmitis.

### Retinal Function Improved in the Absence of TLR2/4 Activation by SLP During Experimental *Bacillus* Endophthalmitis

**Figure 6:**
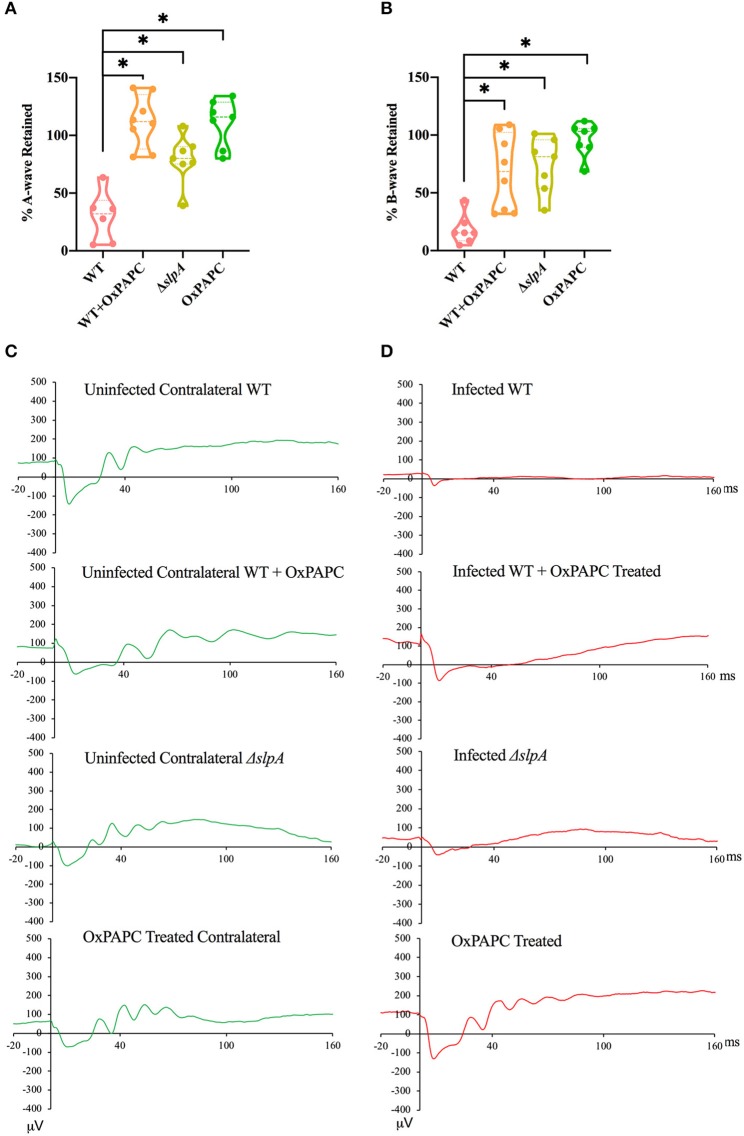
Inhibition of SLP-mediated TLR2/4 activation resulted in significant retention of retinal function during *Bacillus* endophthalmitis. C57BL/6J mouse eyes were injected with 100 CFU WT or Δ*slpA B. thuringiensis*. WT infected and uninfected mouse eyes were treated with 30 ng/μL OxPAPC at 4 h postinfection, and retinal function was assessed by ERG. **(A)** A-wave retention was significantly higher in WT-infected /OxPAPC-treated, Δ*slpA*-infected, and OxPAPC-treated eyes, than WT-infected/untreated eyes. **(B)** B-wave retention was also significantly higher in WT-infected/OxPAPC-treated, Δ*slpA*-infected, and OxPAPC-treated eyes than the WT-infected/untreated eyes. **(C)** Shown are representative waveforms from the uninfected contralateral eyes from each group (green). **(D)** Representative waveforms from WT-infected, WT-infected/OxPAPC-treated, Δ*slpA*-infected, and OxPAPC-treated eyes at 10 h postinfection (red). Values represent the mean ± SEM of the % amplitude retained relative to the contralateral control eye for at least two separate experiments. Data are representative of *N* ≥ 6 eyes; *P < 0.05.

Since the absence of individual TLRs (TLR2 or 4) and their adaptors (MyD88 and TRIF) resulted in retained retinal function in experimental *Bacillus* endophthalmitis (90, 91, 97), we investigated whether inhibition of both TLR2/4 by OxPAPC would have a similar outcome. Analysis of retinal function and the representative waveforms of eyes infected with WT, WT-infected and OxPAPC-treated, Δ*slpA B. thuringiensis*-infected, and OxPAPC-treated only is depicted in Figure 6. The A-wave amplitudes were significantly reduced in WT-infected eyes at 10 h postinfection (*P* < 0.05) to a retained response of ~29%. Compared to WT-infected eyes, WT-infected/OxPAPC-treated, Δ*slpA*-infected, and OxPAPC-treated eyes showed significant retention of retinal function. At 10 h postinfection, the retained response of A-wave function in these groups was ~100% (Figure 6A). The B-wave amplitudes were significantly reduced in the WT *B. thuringiensis*-infected eyes at 10 h postinfection (*P* < 0.05) to a retained response of ~18% (Figure 6B). This response in eyes infected/treated with WT/OxPAPC, Δ*slpA B. thuringiensis*, and OxPAPC was retained to a significantly greater degree compared to that of WT-infected and untreated eyes. The retained responses of B-waves among these groups at 10 h postinfection was ~79%. Representative waveforms demonstrating the differences in A- and B-wave amplitudes of eyes in these groups at 10 h postinfection are shown in Figures 6C,D. Together, these results demonstrated that WT-infected eyes treated with the TLR2/4 inhibitor OxPAPC retained greater retinal function compared to untreated WT *B. thuringiensis*-infected eyes. These results suggested that the activation of TLR2 and TLR4 innate pathways by SLP influenced the loss of retinal function during experimental endophthalmitis.

### Inflammation Was Reduced and Ocular Architecture Was Preserved in the Absence of TLR2/4 Activation by SLP During Experimental *Bacillus* Endophthalmitis

**Figure 7:**
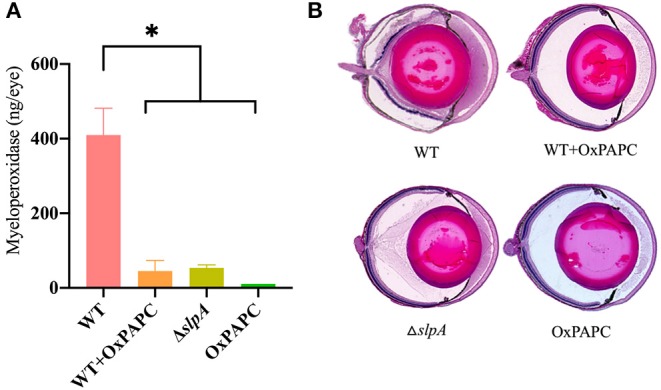
Inhibition of SLP-mediated TLR2/4 activation resulted in reduced inflammatory cell influx and preserved ocular architecture during *Bacillus* endophthalmitis. C57BL/6J mouse eyes were infected with 100 CFU WT or Δ*slpA B. thuringiensis*. Uninfected eyes, or eyes injected with WT *B. thuringiensis* were treated with 30 ng/μL OxPAPC at 4 h postinfection. **(A)** Infiltration of PMN was assessed by quantifying MPO in whole eyes by sandwich ELISA. MPO levels were significantly greater in eyes infected with WT *B. thuringiensis* than in eyes infected with WT and treated with OxPAPC, eyes infected with only Δ*slpA B. thuringiensis*, and eyes treated with OxPAPC alone. Values represent the mean ± SEM of *N* ≥ 4 eyes per group; *P < 0.05. **(B)** Infected and treated eyes were harvested at 10 h postinfection and processed for hematoxylin and eosin staining. Magnification, 10×. Sections are representative of three eyes in each group.

PMN are the primary infiltrating cell type recruited to the site of infection during *Bacillus* endophthalmitis (6, 82, 98). Here, we examined the degree of inflammatory cell influx and retinal damage in WT-infected, WT-infected/OxPAPC-treated, Δ*slpA B. thuringiensis*-infected, and OxPAPC-treated eyes (Figure 7). PMN infiltration in the eye was estimated by quantifying myeloperoxidase (MPO) in eye homogenates. MPO concentrations were significantly greater at 10 h postinfection in WT-infected eyes compared to that of WT-infected/OxPAPC-treated (*P* = 0.0016), Δ*slpA*-infected (*P* = 0.0022), and OxPAPC-treated (*P* = 0.0022) eyes (Figure 7A). The levels of MPO in WT-infected/OXPAPC treated, Δ*slpA*-infected, and OXPAPC-treated eyes were 9-fold, 8-fold, and 38-fold lower compared to that of untreated WT-infected eyes. These results demonstrated that infection with Δ*slpA B. thuringiensis* and inhibition of the TLR2 and TLR4 pathways during experimental endophthalmitis each resulted in reduced MPO levels, indicating less PMN recruitment in these eyes.

A histological comparison of WT-infected, WT-infected/OxPAPC-treated, Δ*slpA*-infected, and OxPAPC-treated eyes is depicted in Figure 7B. At 10 h postinfection, the anterior and posterior segments of WT-infected/OxPAPC-treated, Δ*slpA*-infected, and OxPAPC-treated eyes were similar. The corneas and posterior segments of eyes in these groups had no inflammation and intact retinas. In contrast, untreated eyes infected with WT *B. thuringiensis* had substantial accumulation of infiltrating cells and fibrin in the posterior segment. Corneas in these eyes had significant edema, and retinal layers were detached and often indistinguishable. Together, these findings demonstrated that inhibition of the TLR2 and TLR4 pathways and infection with SlpA-deficient *B. thuringiensis* in experimental endophthalmitis had a similar outcome. In both cases, inflammation was reduced and ocular architecture was preserved. Taken together, these results suggest that SLP contributes to the pathogenesis of *Bacillus* endophthalmitis via TLR2 and TLR4.

## Discussion

The host-pathogen interaction is an early event that dictates the severity and outcome of an infectious disease (116). Although the ocular environment is an immune-privileged site, innate ocular immune defense mechanisms are capable of responding to invading pathogens (74, 75, 77, 78). Ocular defense mechanisms can be easily overwhelmed by infection with a pathogen that cannot be effectively cleared from the eye. *B. cereus* intraocular infection produces a more robust inflammatory response than other ocular bacterial pathogens such as *S. aureus, E. faecalis, S. epidermidis, S. pneumoniae, E. coli*, and *Klebsiella pneumoniae* (7, 73, 82, 117). In *Bacillus* endophthalmitis, within 4 h, PMNs move into the vitreous, and within 8 h into the retinal layers. PMNs not only can disrupt vision through bystander effects on cells in the retina, but their presence in the vitreous can also block the clarity of the visual axis (82, 98). Though *Bacillus* endophthalmitis is a rare intraocular infection, the potential to cause blindness is high, and better therapeutic strategies are needed to improve visual outcomes.

Compared to the envelopes of other Gram-positive intraocular pathogens, the envelope of *Bacillus* contains unique components such as flagella, pili, and a protein coat composed of SLPs (53–55, 60). Flagella aid in the rapid movement of *Bacillus* throughout all parts of the eye, from the initial site of infection into the anterior segment within 6–12 h (89). The absence of motility affected toxin production, and therefore, non-motile *B. cereus* caused less severe disease pathogenesis (19). We also reported that infection with pili-deficient *B. cereus* led to a reduced inflammatory response in the eye, suggesting the importance of pili in that aspect of this disease (93). In a recent report (73), we demonstrated that while the absence of SlpA did not change the growth, cytotoxicity, motility, hemolytic properties, or cell wall composition of *B. thuringiensis*, the absence of SlpA significantly reduced disease severity compared to severe disease caused by the WT parental strain experimental endophthalmitis. SLPs are a major contributor to the pathogenesis of *Bacillus* endophthalmitis (73), but the underlying mechanism by which SLPs contributes to pathogenesis were unknown.

SLPs form para-crystalline protein sheets that assemble on the bacterial surface (56). SLPs and their associated proteins facilitate numerous functions that are critical to cellular physiology and survival (57, 59). A primary function of SLPs are to promote colonization by contributing to the adherence to host tissue (68). It has been reported that SLP of *B. anthracis* helps the pathogen to adhere to HeLa cells, and infection with a SLP-deficient *B. anthracis* resulted in attenuated infection in guinea pigs (118). A recent report suggested that SLP of *Clostridium difficle* played an important role in the colonization to human intestinal epithelial cells by contributing to bacterial adherence (63). We have observed *B. cereus* and *B. thuringiensis* near the inner limiting membrane (ILM) of the retina during experimental endophthalmitis (5, 73). As physical contact between pathogen and the infected tissue is the initial event of any host-pathogen interaction, we compared whether SLPs influenced *Bacillus* adherence to different types of retinal cells. Muller cells are the major retinal cells that span from the outer limiting membrane (OLM) to the ILM, providing structural and hemostasis support (119–122). Since the end feet of retinal Muller cells are located in the ILM, these might be the first retinal cells to encounter pathogens in the posterior segment. RPE cells are important for phototransduction and represent the outer blood retinal barrier. Light-sensitive photoreceptor cells are located anterior to RPE cells (100–103). A recent report suggested that although 661W cells have been used as cone photoreceptor mimics, this cell line expresses markers specific to retinal ganglion cells, such as Rbpms, Brn3b (Pou4f2), Brn3c (Pou4f3), Thy1 and γ-synuclein (Sncg), and thus are retinal ganglion cell-like (99). Here, we demonstrated that SLP plays an important role in mediating *B. thuringiensis* adherence to these cell types *in vitro*, suggesting its role in bacterial attachment to retinal cells.

Evading host defense systems is a key event in successfully establishing an infection. Some organisms are evolutionarily equipped to conceal themselves from the unfriendly environment of the host (123–125). If present, SLPs can protect microorganisms from sudden shifts in pH, exposure to radiation, and changes in mechanical and osmotic stresses. SLPs can also shield bacteria from antimicrobial peptides, lytic enzymes, and bacteriophages (64). It has been reported that the SLP in *Eubacterium yurii* provides resistance to this pathogen against phagocytosis by PMN (72). In addition to providing structural and homeostasis support, Muller cells might also protect the retina by phagocytizing microbes (120, 126, 127). Here, we investigated the role of *B. cereus* SLPs in phagocytosis by human retinal Muller cells, neutrophil like HL-60 cells, and mouse primary neutrophils. We observed significantly less internalized WT *B. thuringiensis* than the Δ*slpA B. thuringiensis* mutant in all three phagocytic cell types. These findings support a role of SLP in promoting *Bacillus* adherence and evading phagocytosis.

Innate immune responses are the host's first line of defense against any invading pathogen, and TLRs are a key mediator in many inflammatory diseases (128, 129). TLRs are critical for initiating an ocular inflammatory response to microbes during keratitis, uveitis, and endophthalmitis (82, 89–91, 95, 130–132). We demonstrated that during *B. cereus* endophthalmitis, TLR2 and TLR4 each directly influenced the severity of intraocular inflammation (90, 91). We also reported the importance of MyD88 and TRIF adaptors in the pathogenesis of *B. cereus* endophthalmitis (97). Here, we demonstrated the activation of TLR2 by the envelope of Gram-positive ocular pathogens, and of these pathogens, only the envelope of *B. thuringiensis* activated TLR4. Since *Bacillus* is Gram-positive bacterium, it possesses several universal TLR2 agonists such as peptidoglycan, lipoteichoic acid, and lipoproteins. However, TLR4 agonists had yet to be identified.

As cell wall-associated proteins, SLPs have the potential to interact with retinal innate receptors. *C. difficile* SLPs have been shown to activate innate and adaptive immunity in a TLR4-dependent manner (133). SLP from *Lactobacillus helveticus* mediated a proinflammatory response through activation of both TLR2 and TLR4 in human macrophages (134). We reported that SLP activated the major transcription factor NF-κb, and induced proinflammatory cytokine production from retinal cells, suggesting that this protein might also activate retinal innate immune pathways (73). Here, by using TLR2 and TLR4 reporter cell lines, we showed for the first time that SLP not only activated TLR2, but also TLR4.

Assessing the role of TLRs and adaptor proteins in *Bacillus* endophthalmitis has been done using specific TLR- or adaptor protein-deficient mice (89–91, 97). Since SLP can signal through both TLR2 and TLR4, we used the oxidized phospholipid OxPAPC to inhibit both pathways (113). OxPAPC competes with CD14, lipid binding protein, and MD2, the accessory proteins that interact with bacterial lipids, and blocks the signaling of both TLR2 and TLR4 (135, 136). A recent report suggested that blocking both TLR2 and TLR4 might lay the foundation for the development of therapies that target inflammasomes during Gram-negative bacterial sepsis (137). OxPAPC has been reported to inhibit LPS (for TLR4) and Pam3Csk4 (for TLR2) ligand-mediated inflammatory responses in mice (138, 139). Anti-inflammatory effects of OxPAPC-directed attenuation of TLR signaling in response to pathogens and pathogen associated molecular patterns (PAMPs) are well recognized (140–142). Here, we showed that OxPAPC dramatically reduced TLR2 and TLR4 activation by their agonists and by *B. cereus* SLP *in vitro*. *In vivo*, we observed an unanticipated reduction in bacterial load in the WT infected-OxPAPC treated group. In contrast, OxPAPC did not alter bacterial growth *in vitro*. We previously reported that absence of TLR2, TLR4, or TLR5 or their adaptor MyD88 did not result in alterations in bacterial burden during *Bacillus* endophthalmitis. But the absence of TRIF, which is a key adaptor for TLR4 signaling, resulted in a significantly reduced bacterial load during *Bacillus* endophthalmitis (89–91, 97). Here, OxPAPC treatment resulted in a reduced bacterial load in the eye from 6- to 12-h postinfection. A greater bacterial burden might be expected in tissue where an inflammatory response is insufficient; however, we did not observe this here. Deficiencies in cathelicidin-related antimicrobial peptide (CRAMP) which led to increased *S. aureus* and *Pseudomonas aeruginosa* burdens in mouse eyes with endophthalmitis and keratitis, respectively, have been reported (143, 144). Hence, it is reasonable to speculate that increased level of AMPs may lead to a lower bacterial burden. However, another report demonstrated that TRIF-deficient mice had low AMP expression levels in the gastrointestinal tract (145), so the physiological involvement of AMPs may be tissue- and infection-specific.

If the inflammation we observed here was ordained exclusively by bacterial burden, infections with WT or Δ*slpA B. thuringiensis* should have resulted in similar levels of inflammation, given that both strains grow similarly in the eye (73). However, the course of inflammation and retinal function loss in WT and Δ*slpA B. thuringiensis* eyes were significantly different, but the rates of bacterial growth in these groups were almost identical (2.2 and 1.99 h^−1^). Since *B. thuringiensis* did not show any growth defects in the presence of increasing concentrations of OxPAPC, the possibility arises that blocking both TLR2 and TLR4 pathways might associated with the upregulation of AMPs in the vitreous. We did not detect expression of AMPs in the retinas of WT or TLR4^−/−^ C57BL/6J mice infected with *Bacillus* at 4 h postinfection (95). Whether OxPAPC has effects other than inhibiting TLR2/4 activation or whether OxPAPC induces expression of AMPs in the retina is an open question.

The retina is a multilayered tissue containing nonregenerative light sensitive cells responsible for biochemical processes involved in proper vision (100). *Bacillus* endophthalmitis destroys these cells, resulting in retinal function loss. We reported that mice lacking individual TLRs (TLR2 or 4) and their adaptors, MyD88 and TRIF, have significant retention of retinal function during *Bacillus* endophthalmitis (89–91, 97). We also reported that infection with a Δ*slpA B. thuringiensis* resulted in better retention of retinal function compared to infection with a WT *B. thuringiensis* (73). Here, we observed that inhibition of both TLR2 and TLR4 signaling with OxPAPC resulted in significantly higher retained retinal function after infection with the WT strain, likely due to the preserved retinal architecture in these eyes. We reported that the absence of SlpA did not change the cytotoxic properties of *Bacillus* or altered its intraocular growth (73). Therefore, it is unlikely that the differences in retinal function loss between untreated WT-infected and WT infected-OxPAPC treated and Δ*slpA* infected eyes were due to variations in toxin production by WT and Δ*slpA B. thuringiensis*. However, the lower bacterial burden in WT infected-OxPAPC treated eyes might have resulted in a reduced cytotoxic effect on the retina which may have been reflected in the retained retinal function in OxPAPC-treated infected eyes.

Retinal detachment is a serious complication of endophthalmitis and has been reported to occur in 4–21% of cases (146). Retinal detachments, folds, and complete dissolution of retinal layers are common in severe cases of endophthalmitis (5, 17, 47). During *B. cereus* endophthalmitis, the absence of TLR2 and TLR4 in mice resulted in less infiltration of PMNs and fibrin accumulation, and preserved retinal architecture (90, 91). The lack of inflammation and intact retinal layers were similar to those reported in infected MyD88^−/−^ and TRIF^−/−^ eyes at 8 and 12 h postinfection (97). Here, we blocked the SLP-mediated TLR2 and TLR4 activation by OxPAPC and observed better preserved retinal architecture in the WT-infected/OxPAPC-treated and Δ*slpA-*infected groups relative to the untreated WT-infected group. We also observed elevated levels of MPO in the untreated group as compared to the treated and Δ*slpA-*infected groups. This suggests that TLR2/4 activation by SLP triggered the TLR2/4 pathways which resulted in the migration of PMNs to the eye and possibly bystander damage to the retina.

Our findings demonstrate for the first time that *Bacillus* SLP impacted endophthalmitis pathogenesis by activating both TLR2 and TLR4 pathways. In addition to identifying SLP as an unexpected TLR4 agonist, we revealed for the first time that inhibiting SLP-mediated TLR2/4 activation in experimental endophthalmitis could reduce disease severity. In *Bacillus* endophthalmitis, treatment failures are frequent despite prompt antibiotic, anti-inflammatory, and surgical intervention. Up to two-thirds of patients with *Bacillus* endophthalmitis lose significant vision, experiencing rapid inflammation and intraocular tissue damage that may also result in the need for enucleation (16, 17). As the number of cataract surgeries and intravitreal injections for degenerative retinal diseases continue to rise, the risk of endophthalmitis will also increase (147–150). Since TLRs and their adaptor proteins are major contributors to the initiation of potentially damaging inflammation in the eye, finding ligands that activate this pathway could be beneficial in formulating plausible strategies for therapeutic intervention to prevent vision loss in endophthalmitis caused by *Bacillus* and other bacterial pathogens.

## Data Availability Statement

The datasets generated for this study are available on request from the corresponding author.

## Ethics Statement

The animal study was reviewed and approved by the Institutional Animal Care and Use Committee of the University of Oklahoma Health Sciences Center (protocol numbers 15-103 and 18-043). All animal experiments were performed in strict accordance with the recommendations in the Guide for the Care and Use of Laboratory Animals of the National Institute of Health and the Association for Research in Vision and Ophthalmology Statement for the Use of Animals in Ophthalmic and Vision Research.

## Author Contributions

MM and MC conceived and designed the experiments. FM, PC, and MC supervised and coordinated the work. AF-M provided the HL-60 cell line. MM performed most experiments and analyzed data. EL and RA provided technical assistance. MM drafted the manuscript. MM, FM, and MC reviewed, edited, and finalized the manuscript.

### Conflict of Interest

The authors declare that the research was conducted in the absence of any commercial or financial relationships that could be construed as a potential conflict of interest.

## References

[1] Mahabadi N Ngando IA Czyz CN Bacterial Endophthalmitis. StatPearls: Treasure Island, FL (2019).

[2] Relhan N Forster RK Flynn HW Jr. Endophthalmitis: then and now. Am J Ophthalmol. (2018) 187:xx–xxvii. 10.1016/j.ajo.2017.11.021 29217351 PMC5873969

[3] Ness T. Endophthalmitis. Ophthalmologe. (2018) 115:697–706. 10.1007/s00347-018-0729-6 29802422

[4] Teweldemedhin M Gebreyesus H Atsbaha AH Asgedom SW Saravanan M. Bacterial profile of ocular infections: a systematic review. BMC Ophthalmol. (2017) 17:212. 10.1186/s12886-017-0612-2 29178851 PMC5702129

[5] Callegan MC Booth MC Jett BD Gilmore MS. Pathogenesis of gram-positive bacterial endophthalmitis. Infect Immun. (1999) 67:3348–56. 10.1128/IAI.67.7.3348-3356.1999 10377112 PMC116517

[6] Astley RA Coburn PS Parkunan SM Callegan MC. Modeling intraocular bacterial infections. Prog Retin Eye Res. (2016) 54:30–48. 10.1016/j.preteyeres.2016.04.007 27154427 PMC4992594

[7] Parkunan SM The pathogenesis of bacterial endophthalmitis. In: Durand ML, Miller JW, Young LHY, editors. Endophthalmitis. Springer International Publishing (2016). p. 17–47.

[8] El Chehab H Renard JP Dot C. Post-traumatic endophthalmitis. J Fr Ophtalmol. (2016) 39:98–106. 10.1016/j.jfo.2015.08.005 26563842

[9] Essex RW Yi Q Charles PG Allen PJ. Post-traumatic endophthalmitis. Ophthalmology. (2004) 111:2015–22. 10.1016/j.ophtha.2003.09.041 15522366

[10] Huber-Spitzy V Grabner G Haddad R Haselberger C. Post-traumatic endophthalmitis caused by *Bacillus cereus*. Klin Monbl Augenheilkd. (1986) 188:52–4. 10.1055/s-2008-1050574 3083151

[11] Bhagat N Li X Zarbin MA. Post-traumatic Endophthalmitis. In: Durand ML, editor. Endophthalmitis. Springer (2016). p. 151–70. 10.1007/978-3-319-29231-1_921397289

[12] Rahmani S Eliott D. Postoperative Endophthalmitis: a review of risk factors, prophylaxis, incidence, microbiology, treatment, and outcomes. Semin Ophthalmol. (2018) 33:95–101. 10.1080/08820538.2017.1353826 29172849

[13] Davidson SI. Post-operative bacterial endophthalmitis. Trans Ophthalmol Soc UK. (1985) 104(Pt 3):278–84. 3875169

[14] Hanscom TA. Postoperative endophthalmitis. Clin Infect Dis. (2004) 38:542–6. 10.1086/381262 14765348

[15] Cunningham ET Flynn HW Relhan N Zierhut M. Endogenous Endophthalmitis. Ocul Immunol Inflamm. (2018) 26:491–95. 10.1080/09273948.2018.1466561 29768116 PMC6448583

[16] Callegan MC Engelbert M Parke DW II Jett BD Gilmore MS. Bacterial endophthalmitis: epidemiology, therapeutics, and bacterium-host interactions. Clin Microbiol Rev. (2002) 15:111–24. 10.1128/CMR.15.1.111-124.2002 11781270 PMC118063

[17] Callegan MC Gilmore MS Gregory M Ramadan RT Wiskur BJ Moyer AL. Bacterial endophthalmitis: therapeutic challenges and host-pathogen interactions. Prog Retin Eye Res. (2007) 26:189–203. 10.1016/j.preteyeres.2006.12.001 17236804 PMC1941835

[18] Callegan MC Kane ST Cochran DC Gilmore MS. Molecular mechanisms of *Bacillus* endophthalmitis pathogenesis. DNA Cell Biol. (2002) 21:367–73. 10.1089/10445490260099647 12167238

[19] Callegan MC Kane ST Cochran DC Novosad B Gilmore MS Gominet M. *Bacillus* endophthalmitis: roles of bacterial toxins and motility during infection. Invest Ophthalmol Vis Sci. (2005) 46:3233–8. 10.1167/iovs.05-0410 16123424

[20] Roy M Chen JC Miller M Boyaner D Kasner O Edelstein E. Epidemic *Bacillus* endophthalmitis after cataract surgery I: acute presentation and outcome. Ophthalmology. (1997) 104:1768–72. 10.1016/S0161-6420(97)30028-1 9373105

[21] Le Saux N Harding GK. *Bacillus cereus* endophthalmitis. Can J Surg. (1987) 30:28–9. 3102030

[22] Rommel CT Kaplan SJ. Post-traumatic *Bacillus cereus* endophthalmitis. Trans Pa Acad Ophthalmol Otolaryngol. (1986) 38:311–4. 3094202

[23] Rasko DA Altherr MR Han CS Ravel J. Genomics of the *Bacillus cereus* group of organisms. FEMS Microbiol Rev. (2005) 29:303–29. 10.1016/j.femsre.2004.12.005 15808746

[24] McDowell RH Friedman H *Bacillus cereus* StatPearls: Treasure Island, FL (2018). 29083665

[25] Peker E Cagan E Dogan M Kilic A Caksen H Yesilmen O. Periorbital cellulitis caused by *Bacillus thuringiensis*. Eur J Ophthalmol. (2010) 20:243–5. 10.1177/112067211002000139 19882531

[26] David DB Kirkby GR Noble BA. *Bacillus cereus* endophthalmitis. Br J Ophthalmol. (1994) 78:577–80. 10.1136/bjo.78.7.577 7918273 PMC504868

[27] Awan KJ. Bacillus endophthalmitis. Ophthalmic Surg. (1992) 23:368. 1603547

[28] Kumar N Garg N Kumar N Van Wagoner N. *Bacillus cereus* panophthalmitis associated with injection drug use. Int J Infect Dis. (2014) 26:165–6. 10.1016/j.ijid.2014.01.019 25016038

[29] Cowan CL Jr Madden WM Hatem GF Merritt JC. Endogenous *Bacillus cereus* panophthalmitis. Ann Ophthalmol. (1987) 19:65–8. 3105407

[30] Grossniklaus H Bruner WE Frank KE Purnell EW. *Bacillus cereus* panophthalmitis appearing as acute glaucoma in a drug addict. Am J Ophthalmol. (1985) 100:334–5. 10.1016/0002-9394(85)90809-8 3927739

[31] Shamsuddin D Tuazon CU Levy C Curtin J. *Bacillus cereus* panophthalmitis: source of the organism. Rev Infect Dis. (1982) 4:97–103. 10.1093/clinids/4.1.97 6803328

[32] Hatem G Merritt JC Cowan CL Jr. *Bacillus cereus* panophthalmitis after intravenous heroin. Ann Ophthalmol. (1979) 11:431–40. 110208

[33] Slean GR Shorstein NH Liu L Paschal JF Winthrop KL Herrinton LJ. Pathogens and antibiotic sensitivities in endophthalmitis. Clin Exp Ophthalmol. (2017) 45:481–88. 10.1111/ceo.12910 28013528 PMC5483388

[34] Baker AS Hemady R. *Bacillus*-induced endophthalmitis. Br J Ophthalmol. (1991) 75:255. 10.1136/bjo.75.4.255 2021602 PMC1042341

[35] Lam KC. Endophthalmitis caused by *Bacillus cereus*: a devastating ophthalmological emergency. Hong Kong Med J. (2015) 21:475.e1–2. 10.12809/hkmj154526 26493084

[36] Miller JJ Scott IU Flynn HW Jr Smiddy WE Murray TG Berrocal A. Endophthalmitis caused by *Bacillus* species. Am J Ophthalmol. (2008) 145:883–8. 10.1016/j.ajo.2007.12.026 18295182

[37] Wang RC Lou PL Ryan EA Kroll AJ. Antibiotic therapy in post-operative endophthalmitis. Semin Ophthalmol. (2002) 17:153–61. 10.1076/soph.17.3.153.14787 12759845

[38] Pan Q Liu Y Wang R Chen T Yang Z Deng Y. Treatment of *Bacillus cereus* endophthalmitis with endoscopy-assisted vitrectomy. Medicine. (2017) 96:e8701. 10.1097/MD.0000000000008701 29390262 PMC5815674

[39] Sakalar YB Ozekinci S Celen MK Treatment of experimental *Bacillus cereus* endophthalmitis using intravitreal moxifloxacin with or without dexamethasone. J Ocul Pharmacol Ther. (2011) 27:593–8. 10.1089/jop.2011.0021 21834669

[40] Khera M Pathengay A Jindal A Jalali S Mathai A Pappuru RR. Vancomycin-resistant Gram-positive bacterial endophthalmitis: epidemiology, treatment options, and outcomes. J Ophthalmic Inflamm Infect. (2013) 3:46. 10.1186/1869-5760-3-46 23607574 PMC3637534

[41] Callegan MC Cochran DC Kane ST Ramadan RT Chodosh J McLean C. Virulence factor profiles and antimicrobial susceptibilities of ocular *Bacillus* isolates. Curr Eye Res. (2006) 31:693–702. 10.1080/02713680600850963 16966141

[42] Shivaramaiah HS Relhan N Pathengay A Mohan N Flynn HW Jr. Endophthalmitis caused by gram-positive bacteria resistant to vancomycin: clinical settings, causative organisms, antimicrobial susceptibilities, and treatment outcomes. Am J Ophthalmol Case Rep. (2018) 10:211–14. 10.1016/j.ajoc.2018.02.030 29552670 PMC5854869

[43] Relhan N Albini TA Pathengay A Kuriyan AE Miller D Flynn HW. Endophthalmitis caused by Gram-positive organisms with reduced vancomycin susceptibility: literature review and options for treatment. Br J Ophthalmol. (2016) 100:446–52. 10.1136/bjophthalmol-2015-307722 26701686 PMC5541392

[44] Wiskur BJ Robinson ML Farrand AJ Novosad BD Callegan MC. Toward improving therapeutic regimens for *Bacillus* endophthalmitis. Invest Ophthalmol Vis Sci. (2008) 49:1480–7. 10.1167/iovs.07-1303 18385066 PMC2531288

[45] Vahey JB Flynn HW Jr. Results in the management of *Bacillus* endophthalmitis. Ophthalmic Surg. (1991) 22:681–6. 1792034

[46] Callegan MC Cochran DC Kane ST Gilmore MS Gominet M Lereclus D. Contribution of membrane-damaging toxins to *Bacillus* endophthalmitis pathogenesis. Infect Immun. (2002) 70:5381–9. 10.1128/IAI.70.10.5381-5389.2002 12228262 PMC128340

[47] Callegan MC Jett BD Hancock LE Gilmore MS. Role of hemolysin BL in the pathogenesis of extraintestinal *Bacillus cereus* infection assessed in an endophthalmitis model. Infect Immun. (1999) 67:3357–66. 10.1128/IAI.67.7.3357-3366.1999 10377113 PMC116518

[48] Declerck N Bouillaut L Chaix D Rugani N Slamti L Hoh F. Structure of PlcR: Insights into virulence regulation and evolution of quorum sensing in Gram-positive bacteria. Proc Natl Acad Sci U.S.A. (2007) 104:18490–5. 10.1073/pnas.0704501104 17998541 PMC2141804

[49] Callegan MC Kane ST Cochran DC Gilmore MS Gominet M Lereclus D. Relationship of plcR-regulated factors to *Bacillus* endophthalmitis virulence. Infect Immun. (2003) 71:3116–24. 10.1128/IAI.71.6.3116-3124.2003 12761089 PMC155772

[50] Agaisse H Gominet M Okstad OA Kolsto AB Lereclus D. PlcR is a pleiotropic regulator of extracellular virulence factor gene expression in *Bacillus thuringiensis*. Mol Microbiol. (1999) 32:1043–53. 10.1046/j.1365-2958.1999.01419.x 10361306

[51] Gohar M Faegri K Perchat S Ravnum S Okstad OA Gominet M. The PlcR virulence regulon of *Bacillus cereus*. PLoS ONE. (2008) 3:e2793. 10.1371/journal.pone.0002793 18665214 PMC2464732

[52] Fouet A Mesnage S. *Bacillus anthracis* cell envelope components. Curr Top Microbiol Immunol. (2002) 271:87–113. 10.1007/978-3-662-05767-4_5 12224525

[53] Silhavy TJ Kahne D Walker S. The bacterial cell envelope. Cold Spring Harb Perspect Biol. (2010) 2:a000414. 10.1101/cshperspect.a000414 20452953 PMC2857177

[54] Siegel SD Liu J Ton-That H. Biogenesis of the Gram-positive bacterial cell envelope. Curr Opin Microbiol. (2016) 34:31–37. 10.1016/j.mib.2016.07.015 27497053 PMC5164837

[55] Dufresne K Paradis-Bleau C. Biology and assembly of the bacterial envelope. Adv Exp Med Biol. (2015) 883:41–76. 10.1007/978-3-319-23603-2_3 26621461

[56] Sleytr UB Messner P Pum D Sara M. Crystalline bacterial cell surface layers. Mol Microbiol. (1993) 10:911–6. 10.1111/j.1365-2958.1993.tb00962.x 7934867

[57] Fagan RP Fairweather NF. Biogenesis and functions of bacterial S-layers. Nat Rev Microbiol. (2014) 12:211–22. 10.1038/nrmicro3213 24509785

[58] Mignot T Denis B Couture-Tosi E Kolsto AB Mock M Fouet A. Distribution of S-layers on the surface of *Bacillus cereus* strains: phylogenetic origin and ecological pressure. Environ Microbiol. (2001) 3:493–501. 10.1046/j.1462-2920.2001.00220.x 11578310

[59] Gerbino E Carasi P Mobili P Serradell MA Gómez-Zavaglia A. Role of S-layer proteins in bacteria. World J Microbiol Biotechnol. (2015) 31:1877–87. 10.1007/s11274-015-1952-9 26410425

[60] Navarre WW Schneewind O. Surface proteins of Gram-positive bacteria and mechanisms of their targeting to the cell wall envelope. Microbiol Mol Biol Rev. (1999) 63:174–229. 10.1128/MMBR.63.1.174-229.1999 10066836 PMC98962

[61] Hynonen U Palva A. *Lactobacillus* surface layer proteins: structure, function and applications. Appl Microbiol Biotechnol. (2013) 97:5225–43. 10.1007/s00253-013-4962-2 23677442 PMC3666127

[62] Bradshaw WJ Roberts AK Shone CC Acharya KR. The structure of the S-layer of Clostridium difficile. J Cell Commun Signal. (2018) 12:319–31. 10.1007/s12079-017-0429-z 29170885 PMC5842191

[63] Merrigan MM Venugopal A Roxas JL Anwar F Mallozzi MJ Roxas BAP. Surface-layer protein A (SlpA) is a major contributor to host-cell adherence of *Clostridium difficile*. PLoS ONE. (2013) 8:e78404. 10.1371/journal.pone.0078404 24265687 PMC3827033

[64] Beveridge TJ Pouwels PH Sara M Kotiranta A Lounatmaa K Kari K. Functions of S-layers. FEMS Microbiol Rev. (1997) 20:99–149. 10.1111/j.1574-6976.1997.tb00305.x 9276929

[65] Ethapa T Leuzzi R Ng YK Baban ST Adamo R Kuehne SA. Multiple factors modulate biofilm formation by the anaerobic pathogen *Clostridium difficile*. J Bacteriol. (2013) 195:545–55. 10.1128/JB.01980-12 23175653 PMC3554014

[66] Sakakibara J Nagano K Murakami Y Higuchi N Nakamura H Shimozato K. Loss of adherence ability to human gingival epithelial cells in S-layer protein-deficient mutants of *Tannerella forsythensis*. Microbiology. (2007) 153(Pt 3):866–76. 10.1099/mic.0.29275-0 17322207

[67] Zhang W Wang H Liu J Zhao Y Gao K Zhang J. Adhesive ability means inhibition activities for *Lactobacillus* against pathogens and S-layer protein plays an important role in adhesion. Anaerobe. (2013) 22:97–103. 10.1016/j.anaerobe.2013.06.005 23792230

[68] Egelseer E Schocher I Sara M Sleytr UB. The S-layer from *Bacillus stearothermophilus* DSM 2358 functions as an adhesion site for a high-molecular-weight amylase. J Bacteriol. (1995) 177:1444–51. 10.1128/JB.177.6.1444-1451.1995 7533757 PMC176758

[69] Shimotahira N Oogai Y Kawada-Matsuo M Yamada S Fukutsuji K Nagano K. The surface layer of *Tannerella forsythia* contributes to serum resistance and oral bacterial coaggregation. Infect Immun. (2013) 81:1198–206. 10.1128/IAI.00983-12 23357386 PMC3639587

[70] Thompson SA. *Campylobacter* surface-layers (S-layers) and immune evasion. Ann Periodontol. (2002) 7:43–53. 10.1902/annals.2002.7.1.43 16013216 PMC2763180

[71] Okuda K Kigure T Yamada S Kaneko T Ishihara K Miura T. Role for the S-layer of *Campylobacter rectus* ATCC33238 in complement mediated killing and phagocytic killing by leukocytes from guinea pig and human peripheral blood. Oral Dis. (1997) 3:113–20. 10.1111/j.1601-0825.1997.tb00022.x 9467352

[72] Kerosuo E Haapasalo M Lounatmaa K. *Eubacterium yurii* subspecies margaretiae is resistant to nonopsonic phagocytic ingestion. Scand J Dent Res. (1993) 101:304–10. 10.1111/j.1600-0722.1993.tb01125.x 8248733

[73] Mursalin MH Coburn PS Livingston E Miller FC Astley R Fouet A. S-layer impacts the virulence of *Bacillus* in Endophthalmitis. Invest Ophthalmol Vis Sci. (2019) 60:3727–39. 10.1167/iovs.19-27453 31479113 PMC6719748

[74] Masli S Vega JL. Ocular immune privilege sites. Methods Mol Biol. (2011) 677:449–58. 10.1007/978-1-60761-869-0>_28 20941626

[75] Streilein JW Ohta K Mo JS Taylor AW. Ocular immune privilege and the impact of intraocular inflammation. DNA Cell Biol. (2002) 21:453–9. 10.1089/10445490260099746 12167248

[76] Sugita S. Role of ocular pigment epithelial cells in immune privilege. Arch Immunol Ther Exp. (2009) 57:263–8. 10.1007/s00005-009-0030-0 19568919

[77] Perez VL Saeed AM Tan Y Urbieta M Cruz-Guilloty F. The eye: a window to the soul of the immune system. J Autoimmun. (2013) 45:7–14. 10.1016/j.jaut.2013.06.011 23871641

[78] Benhar I London A Schwartz M. The privileged immunity of immune privileged organs: the case of the eye. Front Immunol. (2012) 3:296. 10.3389/fimmu.2012.00296 23049533 PMC3448293

[79] Stewart EA Wei R Branch MJ Sidney LE Amoaku WM. Expression of Toll-like receptors in human retinal and choroidal vascular endothelial cells. Exp Eye Res. (2015) 138:114–23. 10.1016/j.exer.2015.06.012 26091789

[80] Chang JH McCluskey PJ Wakefield D. Toll-like receptors in ocular immunity and the immunopathogenesis of inflammatory eye disease. Br J Ophthalmol. (2006) 90:103–8. 10.1136/bjo.2005.072686 16361678 PMC1856909

[81] Kumar A Yu FS. Toll-like receptors and corneal innate immunity. Curr Mol Med. (2006) 6:327–37. 10.2174/156652406776894572 16712478 PMC2666391

[82] Miller FC Coburn PS Huzzatul MM LaGrow AL Livingston E Callegan MC. Targets of immunomodulation in bacterial endophthalmitis. Prog Retin Eye Res. (2019) 73:100763. 10.1016/j.preteyeres.2019.05.004 31150824 PMC6881541

[83] Lin X Fang D Zhou H Su SB. The expression of Toll-like receptors in murine Muller cells, the glial cells in retina. Neurol Sci. (2013) 34:1339–46. 10.1007/s10072-012-1236-1 23207548 PMC3747325

[84] Singh PK Kumar A. Retinal photoreceptor expresses toll-like receptors (TLRs) and elicits innate responses following TLR ligand and bacterial challenge. PLoS ONE. (2015) 10:e0119541. 10.1371/journal.pone.0119541 25767877 PMC4358976

[85] Tan RS Ho B Leung BP Ding JL. TLR cross-talk confers specificity to innate immunity. Int Rev Immunol. (2014) 33:443–53. 10.3109/08830185.2014.921164 24911430 PMC4266099

[86] Qureshi S Medzhitov R. Toll-like receptors and their role in experimental models of microbial infection. Genes Immun. (2003) 4:87–94. 10.1038/sj.gene.6363937 12618855

[87] Delneste Y Beauvillain C Jeannin P. Innate immunity: structure and function of TLRs. Med Sci. (2007) 23:67–73. 10.1051/medsci/200723167 17212934

[88] Muzio M Polentarutti N Bosisio D Manoj Kumar PP Mantovani A. Toll-like receptor family and signalling pathway. Biochem Soc Trans. (2000) 28:563–6. 10.1042/bst0280563 11044375

[89] Parkunan SM Astley R Callegan MC. Role of TLR5 and flagella in *Bacillus* intraocular infection. PLoS ONE. (2014) 9:e100543. 10.1371/journal.pone.0100543 24959742 PMC4068998

[90] Novosad BD Astley RA Callegan MC. Role of Toll-like receptor (TLR) 2 in experimental *Bacillus cereus* endophthalmitis. PLoS ONE. (2011) 6:e28619. 10.1371/journal.pone.0028619 22163046 PMC3232239

[91] Parkunan SM Randall CB Coburn PS Astley RA Staats RL Callegan MC. Unexpected roles for toll-like receptor 4 and TRIF in intraocular infection with Gram-positive bacteria. Infect Immun. (2015) 83:3926–36. 10.1128/IAI.00502-15 26195555 PMC4567632

[92] Mesnage S Haustant M Fouet A. A general strategy for identification of S-layer genes in the *Bacillus cereus* group: molecular characterization of such a gene in *Bacillus thuringiensis* subsp. galleriae NRRL 4045. Microbiology. (2001) 147(Pt 5):1343–51. 10.1099/00221287-147-5-1343 11320137

[93] Callegan MC Parkunan SM Randall CB Coburn PS Miller FC LaGrow AL. The role of pili in *Bacillus cereus* intraocular infection. Exp Eye Res. (2017) 159:69–76. 10.1016/j.exer.2017.03.007 28336259 PMC5492386

[94] Coburn PS Miller FC LaGrow AL Land C Mursalin H Livingston E. Disarming pore-forming toxins with biomimetic nanosponges in intraocular infections. mSphere. (2019) 4:e00262-19. 10.1128/mSphere.00262-19 31092603 PMC6520441

[95] Coburn PS Miller FC LaGrow AL Parkunan SM Blake Randall C Staats RL. TLR4 modulates inflammatory gene targets in the retina during *Bacillus cereus* endophthalmitis. BMC Ophthalmol. (2018) 18:96. 10.1186/s12886-018-0764-8 29661181 PMC5902844

[96] Parkunan SM Randall CB Astley RA Furtado GC Lira SA Callegan MC CXCL1, but not IL-6, significantly impacts intraocular inflammation during infection. J Leukoc Biol. (2016) 100:1125–34. 10.1189/jlb.3A0416-173R 27286792 PMC5069095

[97] Parkunan SM Roehrkasse AM Staats RL Callegan MC Role of MyD88-dependent and MyD88-independent pathways in *Bacillus cereus* endophthalmitis. Invest Ophthalmol Vis Sci. (2014) 55:2872–72.

[98] Ramadan RT Ramirez R Novosad BD Callegan MC. Acute inflammation and loss of retinal architecture and function during experimental *Bacillus* endophthalmitis. Curr Eye Res. (2006) 31:955–65. 10.1080/02713680600976925 17114121

[99] Sayyad Z Sirohi K Radha V Swarup G. 661W is a retinal ganglion precursor-like cell line in which glaucoma-associated optineurin mutants induce cell death selectively. Sci Rep. (2017) 7:16855. 10.1038/s41598-017-17241-0 29203899 PMC5715133

[100] Masland RH. The neuronal organization of the retina. Neuron. (2012) 76:266–80. 10.1016/j.neuron.2012.10.002 23083731 PMC3714606

[101] Hoon M Okawa H Della Santina L Wong RO. Functional architecture of the retina: development and disease. Prog Retin Eye Res. (2014) 42:44–84. 10.1016/j.preteyeres.2014.06.003 24984227 PMC4134977

[102] Wheway G Nazlamova L Turner D Cross S. 661W photoreceptor cell line as a cell model for studying retinal ciliopathies. Front Genet. (2019) 10:308. 10.3389/fgene.2019.00308 31024622 PMC6459963

[103] Thompson AF Crowe ME Lieven CJ Levin LA. Induction of neuronal morphology in the 661W cone photoreceptor cell line with staurosporine. PLoS ONE. (2015) 10:e0145270. 10.1371/journal.pone.0145270 26684837 PMC4684327

[104] Novakowski KE Loukov D Chawla V Bowdish DM. Bacterial binding, phagocytosis, and killing: measurements using colony forming units. Methods Mol Biol. (2017) 1519:297–309. 10.1007/978-1-4939-6581-6_20 27815888

[105] Calzetti F Tamassia N Arruda-Silva F Gasperini S Cassatella MA. The importance of being “pure” neutrophils. J Allergy Clin Immunol. (2017) 139:352–55.e6. 10.1016/j.jaci.2016.06.025 27567327

[106] Sionov RV Assi S Gershkovitz M Sagiv JY Polyansky L Mishalian I. Isolation and characterization of neutrophils with anti-tumor properties. J Vis Exp. (2015):e52933. 10.3791/52933 26132785 PMC4544930

[107] Royer DJ Zheng M Conrady CD Carr DJJ. Granulocytes in ocular HSV-1 infection: opposing roles of mast cells and neutrophils. Invest Ophthalmol Vis Sci. (2015) 56:3763–75. 10.1167/iovs.15-16900 26066745 PMC4468912

[108] Birnie GD. The HL60 cell line: a model system for studying human myeloid cell differentiation. Br J Cancer Suppl. (1988) 9:41–45. 3076064 PMC2149104

[109] Martin SJ Bradley JG Cotter TG. HL-60 cells induced to differentiate towards neutrophils subsequently die via apoptosis. Clin Exp Immunol. (1990) 79:448–53. 10.1111/j.1365-2249.1990.tb08110.x 2317949 PMC1534969

[110] Zucker RM Whittington K Price BJ. Differentiation of HL-60 cells: cell volume and cell cycle changes. Cytometry. (1983) 3:414–8. 10.1002/cyto.990030605 6574016

[111] Czuprynski CJ Henson PM Campbell PA. Killing of *Listeria monocytogenes* by inflammatory neutrophils and mononuclear phagocytes from immune and nonimmune mice. J Leukoc Biol. (1984) 35:193–208. 10.1002/jlb.35.2.193 6423748

[112] Okugawa S Moayeri M Pomerantsev AP Sastalla I Crown D Gupta PK. Lipoprotein biosynthesis by prolipoprotein diacylglyceryl transferase is required for efficient spore germination and full virulence of *Bacillus anthracis*. Mol Microbiol. (2012) 83:96–109. 10.1111/j.1365-2958.2011.07915.x 22103323 PMC3245379

[113] Walton KA Cole AL Yeh M Subbanagounder G Krutzik SR Modlin RL. Specific phospholipid oxidation products inhibit ligand activation of toll-like receptors 4 and 2. Arterioscler Thromb Vasc Biol. (2003) 23:1197–203. 10.1161/01.ATV.0000079340.80744.B8 12775576

[114] Haile LA Polumuri SK Rao R Kelley-Baker L Kryndushkin D Rajaiah R. Cell based assay identifies TLR2 and TLR4 stimulating impurities in Interferon beta. Sci Rep. (2017) 7:10490. 10.1038/s41598-017-09981-w 28874687 PMC5585229

[115] LaGrow AL Coburn PS Miller FC Land C Parkunan SM Luk BT. A novel biomimetic nanosponge protects the retina from the *Enterococcus faecalis* cytolysin. mSphere. (2017) 2:e00335-17. 10.1128/mSphere.00335-17 29202038 PMC5700372

[116] Motley ST Morrow BJ Liu X Dodge IL Vitiello A Ward CK. Simultaneous analysis of host and pathogen interactions during an *in vivo* infection reveals local induction of host acute phase response proteins, a novel bacterial stress response, and evidence of a host-imposed metal ion limited environment. Cell Microbiol. (2004) 6:849–65. 10.1111/j.1462-5822.2004.00407.x 15272866

[117] Astley R Miller FC Mursalin MH Coburn PS Callegan MC. An eye on *Staphylococcus aureus* toxins: roles in ocular damage and inflammation. Toxins. (2019) 11:E356. 10.3390/toxins11060356 31248125 PMC6628431

[118] Kern J Schneewind O. BslA, the S-layer adhesin of *B. anthracis*, is a virulence factor for anthrax pathogenesis. Mol Microbiol. (2010) 75:324–32. 10.1111/j.1365-2958.2009.06958.x 19906175 PMC2828814

[119] Reichenbach A Bringmann A. New functions of Muller cells. Glia. (2013) 61:651–78. 10.1002/glia.22477 23440929

[120] Newman E Reichenbach A. The Muller cell: a functional element of the retina. Trends Neurosci. (1996) 19:307–12. 10.1016/0166-2236(96)10040-0 8843598

[121] Lindqvist N Liu Q Zajadacz J Franze K Reichenbach A. Retinal glial (Müller) cells: sensing and responding to tissue stretch. Invest Ophthalmol Vis Sci. (2010) 51:1683–90. 10.1167/iovs.09-4159 19892866

[122] Bringmann A Wiedemann P. Müller glial cells in retinal disease. Ophthalmologica. (2012) 227:1–19. 10.1159/000328979 21921569

[123] Reddick LE Alto NM. Bacteria fighting back: how pathogens target and subvert the host innate immune system. Mol Cell. (2014) 54:321–28. 10.1016/j.molcel.2014.03.010 24766896 PMC4023866

[124] Barth K Remick DG Genco CA. Disruption of immune regulation by microbial pathogens and resulting chronic inflammation. J Cell Physiol. (2013) 228:1413–22. 10.1002/jcp.24299 23255141 PMC3995356

[125] Finlay BB McFadden G. Anti-immunology: evasion of the host immune system by bacterial and viral pathogens. Cell. (2006) 124:767–82. 10.1016/j.cell.2006.01.034 16497587

[126] Kumar A Shamsuddin N. Retinal Muller glia initiate innate response to infectious stimuli via toll-like receptor signaling. PLoS ONE. (2012) 7:e29830. 10.1371/journal.pone.0029830 22253793 PMC3253788

[127] Singh PK Shiha MJ Kumar A. Antibacterial responses of retinal Müller glia: production of antimicrobial peptides, oxidative burst and phagocytosis. J Neuroinflamm. (2014) 11:33. 10.1186/1742-2094-11-33 24548736 PMC3937076

[128] Beutler BA. TLRs and innate immunity. Blood. (2009) 113:1399–407. 10.1182/blood-2008-07-019307 18757776 PMC2644070

[129] Takeda K Akira S. Toll-like receptors in innate immunity. Int Immunol. (2005) 17:1–14. 10.1093/intimm/dxh186 15585605

[130] Kaur A Kumar V Singh S Singh J Upadhyay N Datta S. Toll-like receptor-associated keratitis and strategies for its management. 3 Biotech. (2015) 5:611–19. 10.1007/s13205-015-0280-y 28324534 PMC4569616

[131] Yu F-SX Hazlett LD. Toll-like receptors and the eye. Invest Ophthalmol Vis Sci. (2006) 47:1255–63. 10.1167/iovs.05-0956 16565355 PMC2666381

[132] Chang JH McCluskey PJ Wakefield D. Recent advances in Toll-like receptors and anterior uveitis. Clin Exp Ophthalmol. (2012) 40:821–8. 10.1111/j.1442-9071.2012.02797.x 22429223

[133] Ryan A Lynch M Smith SM Amu S Nel HJ McCoy CE. A Role for TLR4 in *Clostridium difficile* infection and the recognition of surface layer proteins. PLoS Pathog. (2011) 7:e1002076. 10.1371/journal.ppat.1002076 21738466 PMC3128122

[134] Taverniti V Stuknyte M Minuzzo M Arioli S De Noni I Scabiosi C. S-layer protein mediates the stimulatory effect of *Lactobacillus helveticus* MIMLh5 on innate immunity. Appl Eviron Microbiol. (2013) 79:1221–31. 10.1128/AEM.03056-12 23220964 PMC3568609

[135] Oskolkova OV Afonyushkin T Preinerstorfer B Bicker W von Schlieffen E Hainzl E. Oxidized phospholipids are more potent antagonists of lipopolysaccharide than inducers of inflammation. J Immunol. (2010) 185:7706–12. 10.4049/jimmunol.0903594 21068406

[136] Bochkov VN Oskolkova OV Birukov KG Levonen AL Binder CJ Stockl J. Generation and biological activities of oxidized phospholipids. Antioxid Redox Signal. (2010) 12:1009–59. 10.1089/ars.2009.2597 19686040 PMC3121779

[137] Chu LH Indramohan M Ratsimandresy RA Gangopadhyay A Morris EP Monack DM. The oxidized phospholipid OxPAPC protects from septic shock by targeting the non-canonical inflammasome in macrophages. Nat Commun. (2018) 9:996. 10.1038/s41467-018-03409-3 29520027 PMC5843631

[138] Bochkov VN Kadl A Huber J Gruber F Binder BR Leitinger N. Protective role of phospholipid oxidation products in endotoxin-induced tissue damage. Nature. (2002) 419:77–81. 10.1038/nature01023 12214235

[139] Erridge C Kennedy S Spickett CM Webb DJ. Oxidized phospholipid inhibition of toll-like receptor (TLR) signaling is restricted to TLR2 and TLR4: roles for CD14, LPS-binding protein, and MD2 as targets for specificity of inhibition. J Biol Chem. (2008) 283:24748–59. 10.1074/jbc.M800352200 18559343 PMC3259833

[140] Ma Z Li J Yang L Mu Y Xie W Pitt B. Inhibition of LPS- and CpG DNA-induced TNF-alpha response by oxidized phospholipids. Am J Physiol Lung Cell Mol Physiol. (2004) 286:L808–16. 10.1152/ajplung.00220.2003 14644758

[141] Nonas S Miller I Kawkitinarong K Chatchavalvanich S Gorshkova I Bochkov VN. Oxidized phospholipids reduce vascular leak and inflammation in rat model of acute lung injury. Am J Respir Crit Care Med. (2006) 173:1130–8. 10.1164/rccm.200511-1737OC 16514111 PMC2662943

[142] Meliton AY Meng F Tian Y Sarich N Mutlu GM Birukova AA. Oxidized phospholipids protect against lung injury and endothelial barrier dysfunction caused by heat-inactivated *Staphylococcus aureus*. Am J Physiol Lung Cell Mol Physiol. (2015) 308:L550–62. 10.1152/ajplung.00248.2014 25575515 PMC4360062

[143] Talreja D Singh PK Kumar A. *In vivo* role of TLR2 and MyD88 signaling in eliciting innate immune responses in *Staphylococcal* Endophthalmitis. Invest Ophthalmol Vis Sci. (2015) 56:1719–32. 10.1167/iovs.14-16087 25678692 PMC4356198

[144] Huang LC Reins RY Gallo RL McDermott AM. Cathelicidin-deficient (Cnlp^−/−^) mice show increased susceptibility to *Pseudomonas aeruginosa* Keratitis. Invest Ophthalmol Vis Sci. (2007) 48:4498–508. 10.1167/iovs.07-0274 17898271 PMC4234056

[145] Kezic J Taylor S Gupta S Planck SR Rosenzweig HL Rosenbaum JT Endotoxin-induced uveitis is primarily dependent on radiation-resistant cells and on MyD88 but not TRIF. J Leukoc Biol. (2011) 90:305–11. 10.1189/jlb.0111036 21610198 PMC3133435

[146] Doft BM Kelsey SF Wisniewski SR. Retinal detachment in the endophthalmitis vitrectomy study. Arch Ophthalmol. (2000) 118:1661–65. 10.1001/archopht.118.12.1661 11115260

[147] Hahn P Yashkin AP Sloan FA. Effect of prior anti-VEGF injections on the risk of retained lens fragments and endophthalmitis after cataract surgery in the elderly. Ophthalmology. (2016) 123:309–15. 10.1016/j.ophtha.2015.06.040 26278863 PMC4724443

[148] Merani R Hunyor AP. Endophthalmitis following intravitreal anti-vascular endothelial growth factor (VEGF) injection: a comprehensive review. Int J Retina Vitreous. (2015) 1:9. 10.1186/s40942-015-0010-y 27847602 PMC5088471

[149] Gollogly HE Hodge DO St Sauver JL Erie JC. Increasing incidence of cataract surgery: population-based study. J Cataract Refr Sur. (2013) 39:1383–89. 10.1016/j.jcrs.2013.03.027 23820302 PMC4539250

[150] Dave VP Pathengay A Schwartz SG Flynn HW Jr. Endophthalmitis following pars plana vitrectomy: a literature review of incidence, causative organisms, and treatment outcomes. Clin Ophthalmol. (2014) 8:2183–88. 10.2147/OPTH.S71293 25382968 PMC4222626

